# Supplementary material for: Spatial Topology Reveals Biologically Distinct Recurrent Motifs in Colorectal Cancer
Source: bioRxiv. 2026 Jul 15:2026.07.09.737584. Preprint. [Version 1] doi: 10.64898/2026.07.09.737584 (PMC13405321; doi:10.64898/2026.07.09.737584)
Supplement: Supplement 1 [file NIHPP2026.07.09.737584v1-supplement-1.pdf]

bioRxiv preprint doi: https://doi.org/10.64898/2026.07.09.737584; this version posted July 15, 2026. The copyright holder for this preprint (which was not certified by peer review) is the author/funder, who has granted bioRxiv a license to display the preprint in perpetuity. It is made available under a CC-BY 4.0 International license.

Sup Fig 1. STORM model stability and robustness validation. (A) UMAP projection of per-cell STORM niche embeddings colored by motif label. (B) The same UMAP projection colored by

bioRxiv preprint doi: https://doi.org/10.64898/2026.07.09.737584; this version posted July 15, 2026. The copyright holder for this preprint (which was not certified by peer review) is the author/funder, who has granted bioRxiv a license to display the preprint in perpetuity. It is made available under a CC-BY 4.0 International license.

patient, used to assess patient-driven or section-driven structure. (C) Spatial graph sanity check showing mean undirected graph degree versus cells per sample; point size and color represent the 99th-percentile edge length, and the annotation reports the total number of isolated nodes. (D) Pairwise Pearson correlation of patient-level motif-frequency profiles across the retained clustering seeds after centroid-based motif-label alignment. (E) Matched centroid cosine similarity across retained clustering seeds after Hungarian alignment to the reference seed. Points show mean cosine similarity, and vertical ranges show lower-tail to median centroid similarity summaries. (F) Leave-one-patient-out generalization, showing cosine similarity between each held-out patient's motif-frequency vector and the mean motif-frequency vector of the training patients. (G) Pairwise embedding similarity matrix across graph-construction and receptive-field sensitivity configurations. Values show mean per-cell Pearson correlations between embedding matrices. (H) One-dimensional sensitivity profiles for k-nearest-neighbor graph size and k-hop inference depth relative to the reference configuration (k = 10, 2-hop). (I) Gene Ontology enrichment bubble plots for ALA.

Sup Fig 2. Sample-level association between DFB tumor-boundary coverage and external CD8+ T-cell access to the tumor core and penalty robustness. Each point represents one specimen, with tile-level measurements averaged within each sample (n = 16). The x-axis shows sample-level mean DFB tumor-boundary coverage, summarizing DFB boundary coverage at the specimen level. (A) Higher mean DFB coverage was associated with a higher mean barrier-crossing fraction by external CD8+ T cells (Spearman $\rho = 0.503$, $P = 0.047$). (B) Higher mean DFB coverage was also associated with an increased mean detour ratio, defined as DFB-penalized path cost divided by unweighted graph distance to the tumor core (Spearman $\rho = 0.618$, $P = 0.011$). Dashed lines indicate linear trend fits for visualization; statistical testing used sample-level Spearman correlation. (C) CD8 geodesic access robustness: penalty sensitivity (1, 3, 5, 8).

Sup Fig 3. EOCRC-minus-AOCRC differential Z heatmap.

Sup Fig 4. STORM-Interpret, an agentic large-language-model pipeline for spatial-motif interpretation, applied to two cancers. (A) Pipeline: STORM ingests a per-motif differential-expression (DEG) table with the disease type and transcript technology, pre-processes it by filtering significant DEGs, ranking genes, and flagging low-count motifs, and interprets each motif with a ReAct[50] agent that iterates Thought → Action → Observation over tools spanning the motif's statistics, an open-world pathway knowledge base (62 built-in panels + 228 offline gene sets; MSigDB Hallmark, PanglaoDB), and live Enrichr queries (CellMarker 2024, PanglaoDB, MSigDB Hallmark, GO BP, Jensen DISEASES). A Reflexion critic and a gene-citation validation step then vet the result, which is exported as JSON, CSV, and HTML. (B) Case study 1, rhabdomyosarcoma (RMS; Xenium, 10 spatial motifs): for each motif, the name, abbreviation, key marker genes, putative primary cell type, number of significant DEGs, confidence score, and gene-citation validation (✓) are listed (9 interpreted; 1 skipped for insufficient DEGs). (C) Case study 2, lung cancer (LUAD+SCLC; Xenium, 5 spatial motifs), additionally listing the number of agent tool steps per motif. (D) Auto-generated interactive HTML report for the RMS run, summarizing pipeline-level metrics (total, interpreted, and skipped motifs; validation pass rate; mean confidence) and per-motif interpretation cards. DEG, differentially expressed gene; padj, Benjamini-Hochberg-adjusted P value.

Sup Table 1. The 10-motif STORM atlas of the colorectal cancer microenvironment.

Sup Table 2. Xenium data detail information.

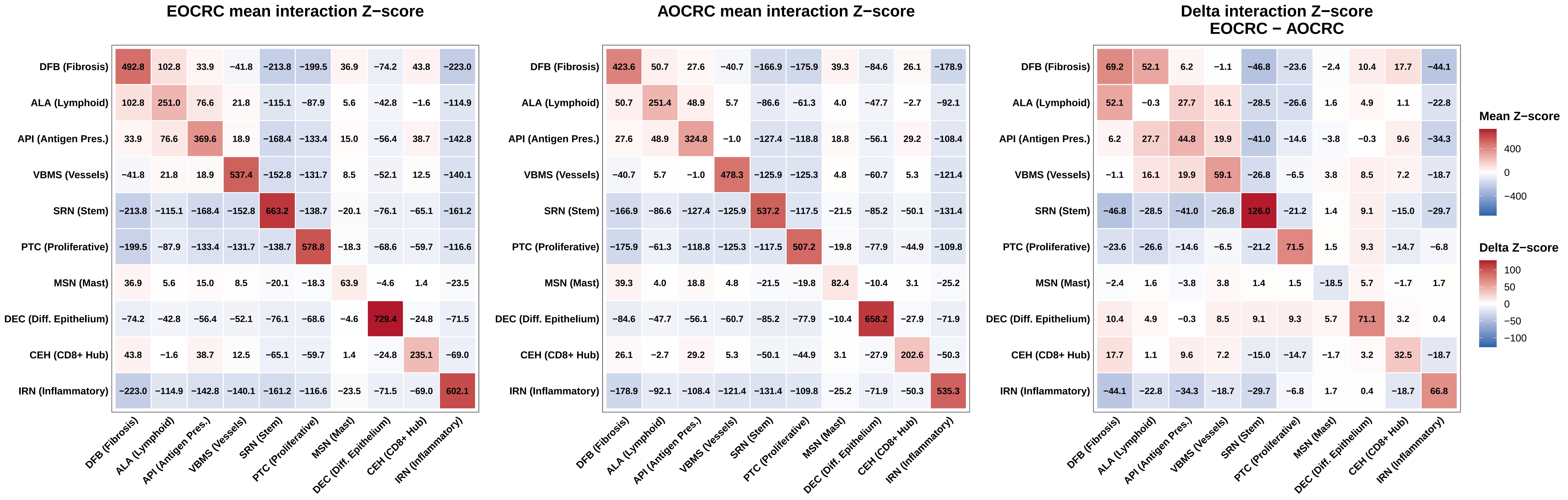
EOCRC mean interaction Z−score
DFB (Fibrosis) 492.8 102.8 33.9 −41.8 −213.8 −199.5 36.9 −74.2 43.8 −223.0
ALA (Lymphoid) 102.8 251.0 76.6 21.8 −115.1 −87.9 5.6 −42.8 −1.6 −114.9
API (Antigen Pres.) 33.9 76.6 369.6 18.9 −168.4 −133.4 15.0 −56.4 38.7 −142.8
VBMS (Vessels) −41.8 21.8 18.9 537.4 −152.8 −131.7 8.5 −52.1 12.5 −140.1
SRN (Stem) −213.8 −115.1 −168.4 −152.8 663.2 −138.7 −20.1 −76.1 −65.1 −161.2
PTC (Proliferative) −199.5 −87.9 −133.4 −131.7 −138.7 578.8 −18.3 −68.6 −59.7 −116.6
MSN (Mast) 36.9 5.6 15.0 8.5 −20.1 −18.3 63.9 −4.6 1.4 −23.5
DEC (Diff. Epithelium) −74.2 −42.8 −56.4 −52.1 −76.1 −68.6 −4.6 729.4 −24.8 −71.5
CEH (CD8+ Hub) 43.8 −1.6 38.7 12.5 −65.1 −59.7 1.4 −24.8 235.1 −69.0
IRN (Inflammatory) −223.0 −114.9 −142.8 −140.1 −161.2 −116.6 −23.5 −71.5 −69.0 602.1
DFB (Fibrosis)
ALA (Lymphoid)
API (Antigen Pres.)
VBMS (Vessels)
SRN (Stem)
PTC (Proliferative)
MSN (Mast)
DEC (Diff. Epithelium)
CEH (CD8+ Hub)
IRN (Inflammatory)
AOCRC mean interaction Z−score
DFB (Fibrosis) 423.6 50.7 27.6 −40.7 −166.9 −175.9 39.3 −84.6 26.1 −178.9
ALA (Lymphoid) 50.7 251.4 48.9 5.7 −86.6 −61.3 4.0 −47.7 −2.7 −92.1
API (Antigen Pres.) 27.6 48.9 324.8 −1.0 −127.4 −118.8 18.8 −56.1 29.2 −108.4
VBMS (Vessels) −40.7 5.7 −1.0 478.3 −125.9 −125.3 4.8 −60.7 5.3 −121.4
SRN (Stem) −166.9 −86.6 −127.4 −125.9 537.2 −117.5 −21.5 −85.2 −50.1 −131.4
PTC (Proliferative) −175.9 −61.3 −118.8 −125.3 −117.5 507.2 −19.8 −77.9 −44.9 −109.8
MSN (Mast) 39.3 4.0 18.8 4.8 −21.5 −19.8 82.4 −10.4 3.1 −25.2
DEC (Diff. Epithelium) −84.6 −47.7 −56.1 −60.7 −85.2 −77.9 −10.4 658.2 −27.9 −71.9
CEH (CD8+ Hub) 26.1 −2.7 29.2 5.3 −50.1 −44.9 3.1 −27.9 202.6 −50.3
IRN (Inflammatory) −178.9 −92.1 −108.4 −121.4 −131.4 −109.8 −25.2 −71.9 −50.3 535.3
DFB (Fibrosis)
ALA (Lymphoid)
API (Antigen Pres.)
VBMS (Vessels)
SRN (Stem)
PTC (Proliferative)
MSN (Mast)
DEC (Diff. Epithelium)
CEH (CD8+ Hub)
IRN (Inflammatory)
Delta interaction Z−score
EOCRC − AOCRC
DFB (Fibrosis) 69.2 52.1 6.2 −1.1 −46.8 −23.6 −2.4 10.4 17.7 −44.1
ALA (Lymphoid) 52.1 −0.3 27.7 16.1 −28.5 −26.6 1.6 4.9 1.1 −22.8
API (Antigen Pres.) 6.2 27.7 44.8 19.9 −41.0 −14.6 −3.8 −0.3 9.6 −34.3
VBMS (Vessels) −1.1 16.1 19.9 59.1 −26.8 −6.5 3.8 8.5 7.2 −18.7
SRN (Stem) −46.8 −28.5 −41.0 −26.8 126.0 −21.2 1.4 9.1 −15.0 −29.7
PTC (Proliferative) −23.6 −26.6 −14.6 −6.5 −21.2 71.5 1.5 9.3 −14.7 −6.8
MSN (Mast) −2.4 1.6 −3.8 3.8 1.4 1.5 −18.5 5.7 −1.7 1.7
DEC (Diff. Epithelium) 10.4 4.9 −0.3 8.5 9.1 9.3 5.7 71.1 3.2 0.4
CEH (CD8+ Hub) 17.7 1.1 9.6 7.2 −15.0 −14.7 −1.7 3.2 32.5 −18.7
IRN (Inflammatory) −44.1 −22.8 −34.3 −18.7 −29.7 −6.8 1.7 0.4 −18.7 66.8
DFB (Fibrosis)
ALA (Lymphoid)
API (Antigen Pres.)
VBMS (Vessels)
SRN (Stem)
PTC (Proliferative)
MSN (Mast)
DEC (Diff. Epithelium)
CEH (CD8+ Hub)
IRN (Inflammatory)
Mean Z−score
400
0
−400
Delta Z−score
100
50
0
−50
−100

bioRxiv preprint doi: https://doi.org/10.64898/2026.07.09.737584; this version posted July 15, 2026. The copyright holder for this preprint (which was not certified by peer review) is the author/funder, who has granted bioRxiv a license to display the preprint in perpetuity. It is made available under a CC-BY 4.0 International license.

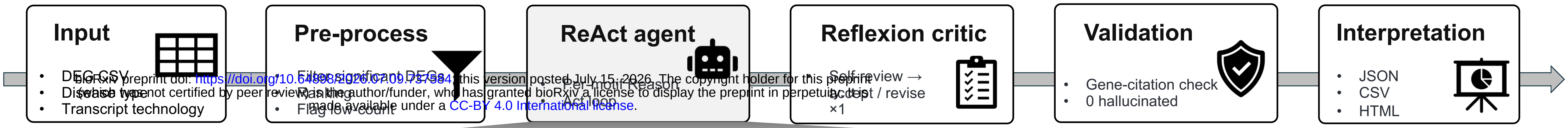

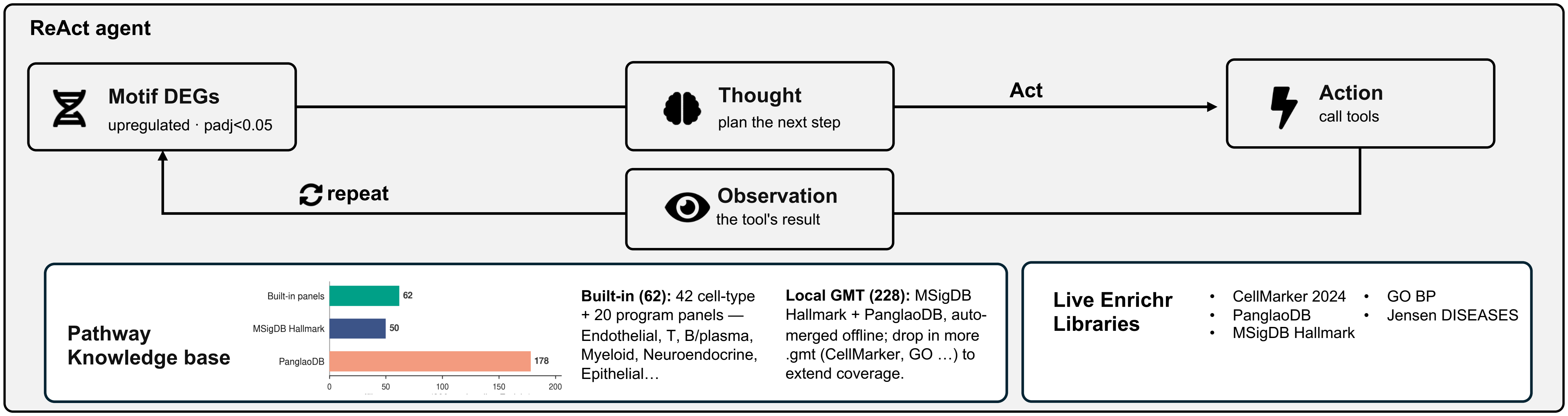

Rhabdomyosarcoma (RMS) · Xenium — 10 spatial motifs

#	Motif name	Abbr	Key marker genes	Primary cell type	DEGs	Conf.	Val.
0	Angiogenic Vascular Niche	AVN	PECAM1, CDH5, PLVAP, TIE1	Endothelial cell	892	0.90	✓
1	Metabolically Active Progenitor	MAP	EEF1G, H19, SLC25A39, BIRC5	Muscle progenitor	81	0.55	✓
2	Myeloid Inflammatory Hub	MIH	CD68, CD14, MSR1, FCGR2A	Tumour-assoc. macrophage	693	0.88	✓
3	Differentiated Myogenic Niche	DMN	TNNT2, RYR1, STAC3, MYO18B	Diff. rhabdomyoblast	39	0.85	✓
4	Stem-like Proliferative Core	SPC	MEGF10, CCND2, SKP2, CD44	Stem-like RMS cell	589	0.68	✓
5	Uninterpretable — Insufficient DEGs	UND	SOX2-OT, F7	— (skipped)	2	—	—
6	Proliferating Tumor Core	PTC	BIRC5, TOP2A, MKI67, CDK1	Proliferating RMS cell	599	0.87	✓
7	Desmoplastic Fibroblast Barrier	DFB	POSTN, COL5A1, FAP, THY1	Cancer-assoc. fibroblast	587	0.89	✓
8	Undifferentiated Myogenic Progenitor	UMP	H19, DLK1, TNNT2, RYR1	Undiff. rhabdomyoblast	42	0.72	✓
9	Immune-Activated Cytotoxic Hub	ICH	CD3E, STAT1, MX1, GZMB	Cytotoxic T cell	729	0.86	✓

Lung cancer (LUAD+SCLC) · Xenium — 5 spatial motifs

#	Motif name	Abbr	Key marker genes	Primary cell type	DEGs	Steps	Conf.	Val.
0	Plasma B lymphoid	PBL	MZB1, POU2AF1, CD79A, MS4A1	B / plasma cells	61	5	0.87	✓
1	Alveolar Epithelial Carcinoma	AEC	EPCAM, KRT8, SLC34A2, NAPSA	Epithelial / carcinoma	191	5	0.89	✓
2	Neuroendocrine Epithelial State	NEES	EPCAM, SYP, RIMS2, PROX1	Neuroendocrine carcinoma	175	7	0.87	✓
3	Immune Stromal Niche	ISN	CD3E, CD8A, CD69, COL1A2	T cells	49	5	0.87	✓
4	Angiogenic Stroma	ANGS	VWF, PLVAP, CD34, FN1	Endothelial cells	67	4	0.89	✓

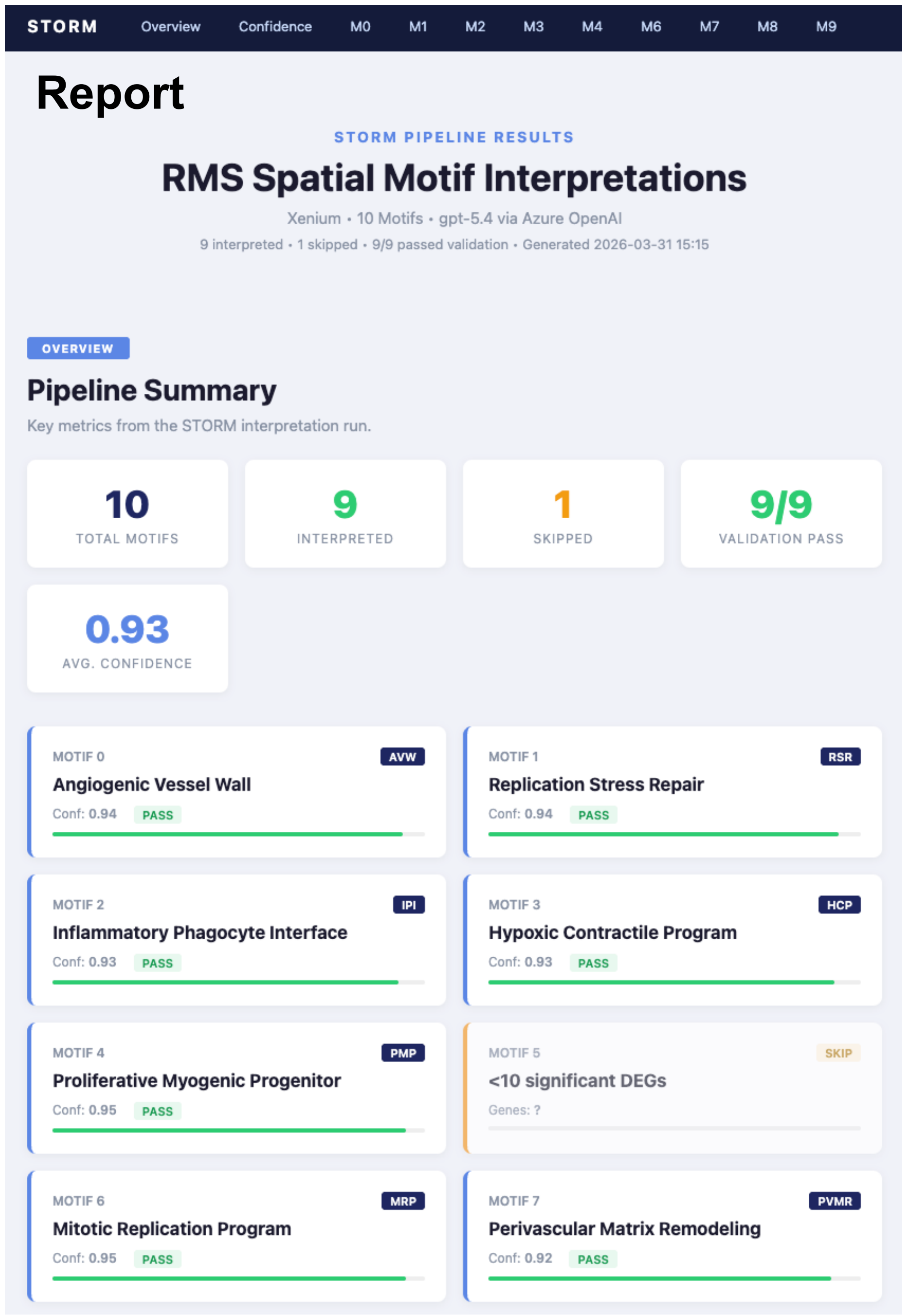

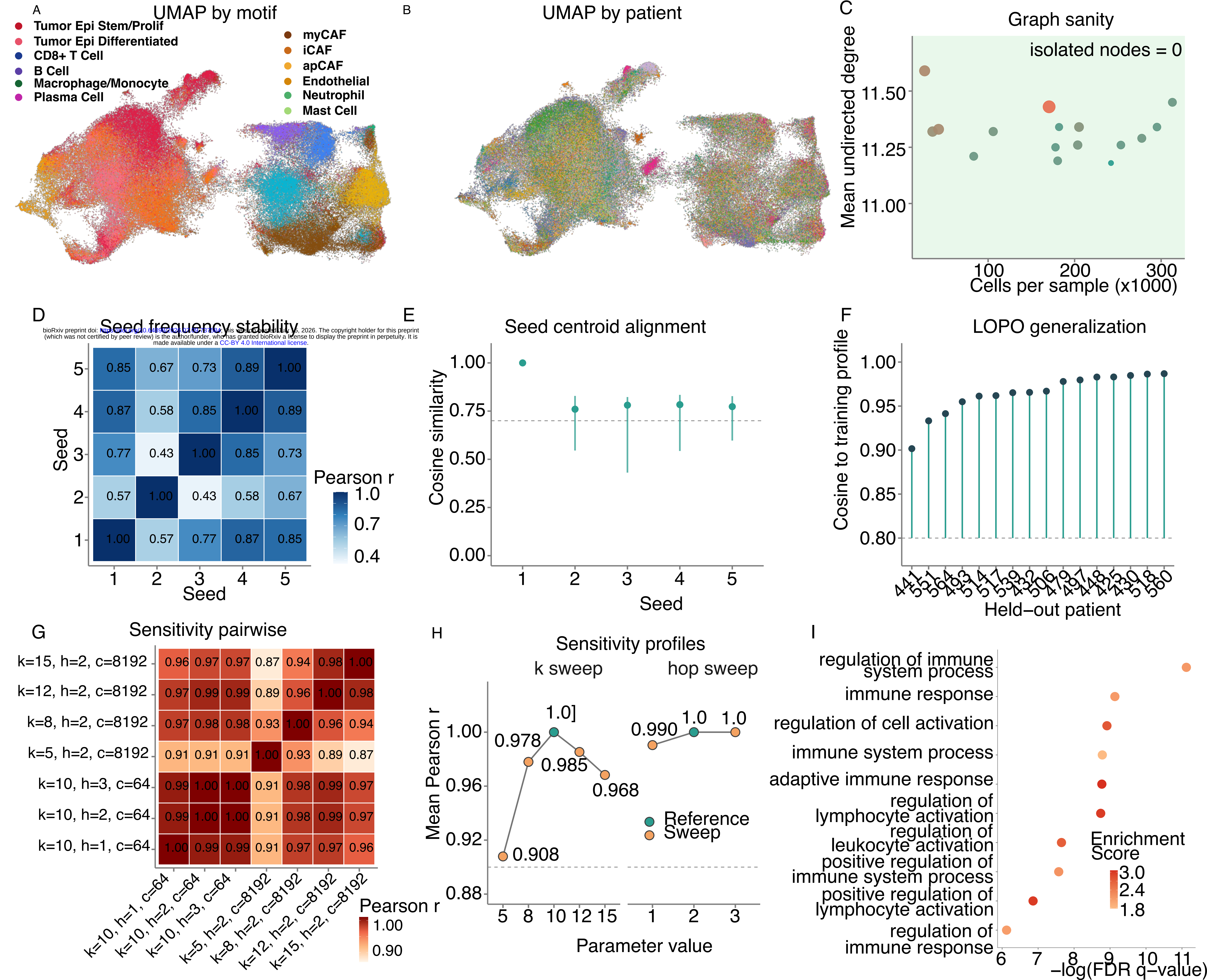

bioRxiv preprint doi: https://doi.org/10.64898/2026.07.09.737584; this version posted July 15, 2026. The copyright holder for this preprint (which was not certified by peer review) is the author/funder, who has granted bioRxiv a license to display the preprint in perpetuity. It is made available under a CC-BY 4.0 International license.

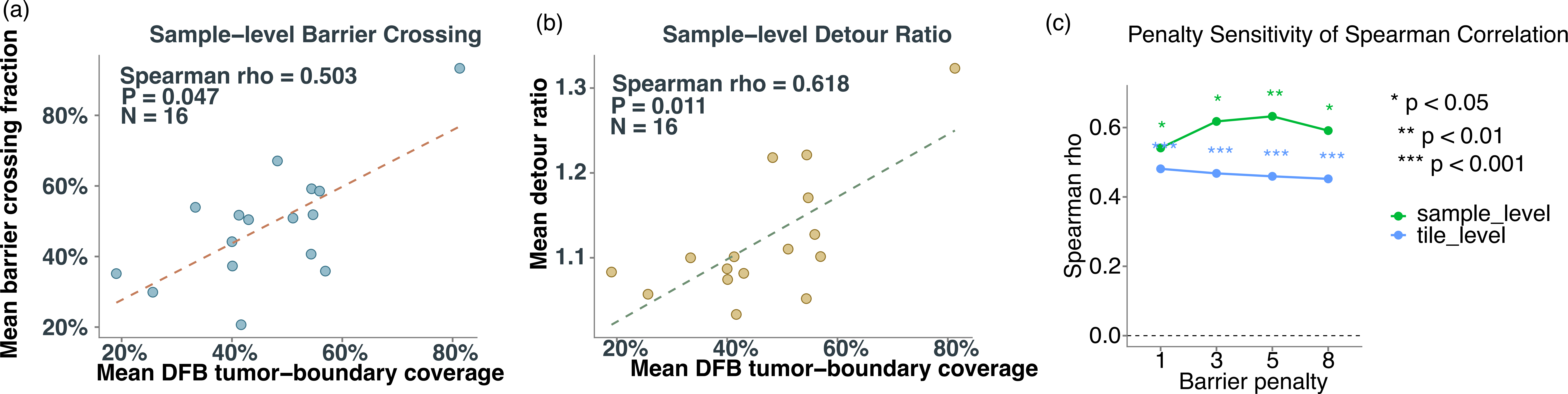
(a)
Sample–level Barrier Crossing
Spearman rho = 0.503
P = 0.047
N = 16
Mean barrier crossing fraction
20%
40%
60%
80%
Mean DFB tumor–boundary coverage
(b)
Sample–level Detour Ratio
Spearman rho = 0.618
P = 0.011
N = 16
Mean detour ratio
1.1
1.2
1.3
Mean DFB tumor–boundary coverage
(c)
Penalty Sensitivity of Spearman Correlation
Spearman rho
0.0
0.2
0.4
0.6
1
3
5
8
Barrier penalty
* p < 0.05
** p < 0.01
*** p < 0.001
sample_level
tile_level
